# Progression-Free Survival and Time to Progression as Potential Surrogate Endpoints for Overall Survival in Chemoradiotherapy Trials in Limited-Stage Small-Cell Lung Cancer: A Systematic Review and Meta-Analysis

**DOI:** 10.3389/fonc.2022.810580

**Published:** 2022-01-28

**Authors:** Yin Yang, Jianyang Wang, Wenqing Wang, Tao Zhang, Jingjing Zhao, Yu Wang, Yexiong Li, Luhua Wang, Nan Bi

**Affiliations:** ^1^ Department of Radiation Oncology, National Cancer Center/National Clinical Research Center for Cancer/Cancer Hospital, Chinese Academy of Medical Sciences and Peking Union Medical College, Beijing, China; ^2^ Department of Radiation Oncology, National Cancer Center/National Clinical Research Center for Cancer/Cancer Hospital & Shenzhen Hospital, Chinese Academy of Medical Sciences and Peking Union Medical College, Shenzhen, China

**Keywords:** limited-stage small-cell lung cancer, surrogate endpoint, overall survival, progression-free survival, time to progression, chemoradiotherapy

## Abstract

**Purpose:**

To investigate whether progression-free survival (PFS) or time to progression (TTP) could be a valid surrogate endpoint for overall survival (OS) in patients with limited-stage small-cell lung cancer (LS-SCLC) receiving combined chemoradiotherapy.

**Methods:**

Literature searching was performed in PubMed, Embase, and The Cochrane Library up to 2021. Prediction models were firstly established using data from phase III randomized controlled trials (RCTs) and then externally validated in phase II and retrospective studies. Correlation analysis was evaluated by a weighted linear regression model at both trial and arm levels. Cross-validation was performed to assess the consistency and robustness of the established models.

**Results:**

37 studies, including 15 phase III RCTs, 12 phase II studies, and 10 retrospective studies, were selected in the final analysis. In trial-level surrogacy, a very good correlation was observed between hazard ratios (HRs) of PFS/TTP and OS (R^2^ = 0.783, 95% CI 0.771–0.794). In arm-level surrogacy, very good correlations were also observed between 2-year (R^2^ = 0.823, 95% CI 0.814–0.832), 3-year (R^2^ = 0.843, 95% CI 0.833–0.850), 5-year (R^2^ = 0.852, 95% CI 0.843–0.859) PFS/TTP, and 5-year OS. An excellent correlation was observed between 4-year PFS/TTP and 5-year OS (R^2^ = 0.906, 95% CI 0.901–0.910). Cross-validation demonstrated reasonable overall consistency. External validation in phase II and retrospective studies showed good agreement (R^2^, 0.728–0.824).

**Conclusions:**

PFS/TTP was a valid surrogate endpoint for OS in patients with LS-SCLC receiving combined chemoradiotherapy. The finding provides high-level evidence to support PFS/TTP as the primary endpoint in clinical trials so as to speed up introducing novel agents to the treatment of LS-SCLC.

## Introduction

Small-cell lung cancer (SCLC) is the most aggressive subtype of lung cancer, with an estimated incidence of 4% and 250,000 cancer deaths worldwide (1, 2). Limited disease accounts for one third of the total cases. Besides the patients with T1–2N0M0 disease (AJCC 8th) who may be surgical candidates, chemoradiotherapy is the standard of care for most of limited-stage small-cell lung cancer (LS-SCLC) (95%) (3) and results in a 5-year overall survival (OS) of 20%–30% (4, 5).

OS is the gold-standard endpoint in randomized controlled trials (RCTs) as it is simple and unbiased. Especially, the 5-year OS rate is commonly used to assess the long-term benefits and toxicities of the treatment. However, using OS as the primary endpoint requires a large number of patients and long-term follow-up, leading to higher costs and delays in introducing novel drugs. Given these disadvantages, using an early surrogate endpoint in RCTs would shorten the time duration and save the research resources. Until now, The Food and Drug Administration has granted accelerated approval of many drugs based on surrogate endpoints of progression-free survival (PFS) or time to progression (TTP). For example, crizotinib was approved for anaplastic lymphoma kinase-positive non-small-cell lung cancer on the basis of PFS (6) and sunitinib for gastrointestinal stromal tumor and renal cell carcinoma on the basis of TTP (7). PFS and TTP have also been demonstrated to be valid surrogate endpoints for OS in some malignancies (8–12). However, an early valid surrogate endpoint has never been reported in LS-SCLC patients.

Reviewing various endpoints in clinical trials of LS-SCLC, PFS and TTP were potential surrogate endpoint for OS (4, 13, 14). Hereby, we investigated whether PFS/TTP could be used as an early efficient surrogate endpoint in LS-SCLC through literature-based analysis at trial and arm-level.

## Literature Search and Study Selection

### Search Strategy

Articles published before December 25, 2021 were identified *via* a systematic literature search of PubMed, Embase, and The Cochrane Library. The keywords were “Limited” and “Small Cell Lung Cancer” and “Chemoradiotherapy”. The search strategy is shown in **Supplementary Table 1**. The database searches were carried out independently by two authors (YY and JY, W).

### Study Selection

The inclusion criteria of studies were as following: (1) LS-SCLC; (2) all patients received chemoradiotherapy but not surgery; (3) phase III RCTs, phase II trials, and retrospective studies; (4) the outcomes of studies include the following endpoints: hazard ratios (HRs) for OS and PFS/TTP, or absolute PFS/TTP rates (1, 2, 3, 4, 5-year) and 5-year OS. (5) English language; (6) at least 30 patients per arm. (7) published after 1990.

We excluded literatures without original data, phase I studies, inadequate survival data, systematic reviews, case reports, and other irrelevant publications.

### Data Extraction

The following information from included studies were extracted: publication year, design, treatments of groups, number of patients, median follow-up time, and endpoints. For phase III RCTs, the endpoints were HRs for OS and PFS/TTP, absolute PFS/TTP rates (1, 2, 3, 4, 5-year) and 5-year OS (**Table 1**). The HRs or survival rates at different time point were obtained from the text or Kaplan–Meier curves, according to methods by Tierney et al. (26). For phase II trials and retrospective studies, the endpoints were absolute PFS/TTP rates (year 1, 2, 3, 4, 5) and 5-year OS (**Table 2**).

**Table 1:** Summary of 15 phase III randomized controlled trials included in the current meta-analysis.

Study	Study period	Treatment arm	Radiotherapy dose	Chemotherapy regimen	No. of patients	Median follow-up, year	OS, %		PFS/TTP, %					
							Hazard ratio	5-year	Hazard ratio	1-year	2-year	3-year	4-year	5-year
Jett, (13)	1979.09–1986.03	With etoposide	37.5 Gy/2.5 Gy/15f, QD	1st, 2nd, 3rd cycle: cyclophosphamide, doxorubicin, vincristine, etoposide. 4th cycle: cyclophosphamide, vincristine, etoposide.	118	NA	0.8*^b^*	13*^a, b^*	0.87*^b^*	40.4*^b^*	23.4*^b^*	18.1*^b^*	16.2*^b^*	13.9*^b^*
		Without etoposide	37.5 Gy/2.5 Gy/15f, QD	1st, 2nd, 3rd cycle: cyclophosphamide, doxorubicin, vincristine. 4th cycle: cyclophosphamide, vincristine.	113			10*^a,b^*		32.4*^b^*	11.8*^b^*	8.8*^b^*	8.8*^b^*	8.8*^b^*
Murray, (15)	1985.01–1988.12	Early RT	40 Gy/15f, QD	1st, 3rd, 5th cycle: cyclophosphamide, doxorubicin, vincristine. 2nd, 4th, 6th cycle: etoposide, cisplatin.	155	5.0	0.79*^b^*	20*^a,b^*	0.85*^b^*	59.7*^b^*	27*^b^*	26*^a, b^*	22.2*^b^*	22.2*^b^*
		Late RT	40 Gy/15f, QD	1st, 3rd, 5th cycle: cyclophosphamide, doxorubicin, vincristine. 2nd, 4th, 6th cycle: etoposide, cisplatin.	153			11*^a,b^*		48*^b^*	23.2*^b^*	19*^a,b^*	16.2*^b^*	16.2*^b^*
Gregor, (16)	1989.03–1995.01	Alternating CRT	50 Gy/2.5 Gy/20f, QD	5 cycles: cyclophosphamide, doxorubicin, etoposide	170	3.6	1.15*^b^*	3.7*^b^*	1.25*^b^*	38.1*^b^*	14.4*^b^*	9.5*^b^*	7*^b^*	7*^b^*
		Sequential CRT	50 Gy/2.5 Gy/20f, QD	5 cycles: cyclophosphamide, doxorubicin, etoposide	165			9.9*^b^*		46.8*^b^*	21.6*^b^*	16.4*^b^*	14.4*^b^*	14.4*^b^*
Turrisi, (4)	1989.05–1992.07	Once-daily RT	45 Gy/1.8 Gy/25f, QD	4 cycles: etoposide, cisplatin	206	8.0	–	16*^a^*	NA	NA	24*^a^*	NA	NA	NA
		Twice-daily RT	45 Gy/1.5 Gy/30f, BID	4 cycles: etoposide, cisplatin	211			26*^a^*		NA	29*^a^*	NA	NA	NA
Takada, (14)	1991.05–1995.01	Sequential CRT	45 Gy/1.5 Gy/30f, BID	4 cycles: etoposide, cisplatin	114	NA	1.22	18.3*^a^*	1.18	36.7	19.4	15.9	15.7	15.5
		Concurrent CRT	45 Gy/1.5 Gy/30f, BID	4 cycles: etoposide, cisplatin	114			23.7*^a^*		49	29	25.5	21.5	18.3
Schild, (17)	1990.09–1996.11	Once-daily RT	50.4 Gy/1.8 Gy/28f, QD	6 cycles: etoposide, cisplatin	131	7.4	1.01	21*^a^*	1.11	51.9	31.3*^a^*	25.3	20.5	19.8*^a^*
		Twice-daily RT	48 Gy/1.5 Gy/32f, BID	6 cycles: etoposide, cisplatin	130			22*^a^*		51.9	30.8*^a^*	27.5	23.6	21*^a^*
Blackstock, (18)	1987.08–1992.11	Continuous RT	50 Gy/2 Gy/25f, QD	1st, 2nd, 5th cycle: cisplatin, etoposide. 3rd, 4th, 6th cycles: cyclophosphamide, vincristine, doxorubicin.	56	12.7	0.98	18*^a^*	1.09	33.8	23.2	18	16.2	16.2
		Split-course RT	50 Gy/2.5 Gy/20f, QD	1st, 2nd, 5th cycle: cisplatin, etoposide. 3rd, 4th, 6th cycles: cyclophosphamide, vincristine, doxorubicin.	54			17*^a^*		40.8	18.6	16.9	12.9	10.7
Giaccone, (19)	1998.03–2002.10	Without Bec2/Bacilli Calmette-Guerin	NA	93% patients received platinum-based chemotherapy	258	3.0	0.89*^a^*	18.5	0.9*^a^*	32.2*^a^*	25.4*^a^*	22.7	19.4	19.4
		With Bec2/Bacilli Calmette-Guerin	NA	93% patients received platinum-based chemotherapy	257			16.5		31.1*^a^*	24.9*^a^*	17.9	15.9	15.9
McClay, (20)	1993.08–1999.01	Without tamoxifen	50 Gy/2 Gy/25f, QD	5 cycles: etoposide, cisplatin	154	4.4	0.99	18.1	0.89	50.7	26.6	23*^a^*	20.8	17.6
		With tamoxifen	50 Gy/2 Gy/25f, QD	5 cycles: etoposide, cisplatin	153			14.3		42.3	24.2	22*^a^*	14.6	11.7
Sculier, (21)	1993.03–2006.03	Standard-dose cisplatin	39.9 Gy/2.66 Gy/15f, QD	6 cycles: etoposide, cisplatin	104	4.5	1.12*^a, b^*	18^a, ^ *^b^*	1.11^a, ^ *^b^*	NA	23*^a, b^*	NA	NA	16*^a,b^*
		High-dose cisplatin	39.9 Gy/2.66 Gy/15f, QD	6 cycles: etoposide, cisplatin	100			21*^a,b^*		NA	26*^a,b^*	NA	NA	19*^a,b^*
Le Péchoux, (22)	1999.09–2005.12	Standard-dose PCI	NA	NA	360	3.3	1.2*^a^*	NA	1.16*^a^*	NA	NA	NA	NA	NA
		High-dose PCI	NA	NA	360			NA		NA	NA	NA	NA	NA
Sun, (23)	2003.07–2010.06	Early RT	52.5 Gy/2.1 Gy/25f, QD	4 cycles: etoposide, cisplatin	111	5.0	0.9*^a^*	24.3*^a^*	1.1*^a^*	51.8*^a^*	28*^a^*	24.2	24.2	24.2
		Late RT	52.5 Gy/2.1 Gy/25f, QD	4 cycles: etoposide, cisplatin	108			24*^a^*		48.1*^a^*	23.5*^a^*	23.5	21	21
Kubota, (24)	2002.09–2006.10	EP chemotherapy	45 Gy/1.5 Gy/30f, BID	4 cycles: etoposide, cisplatin	129	6.3	0.92*^a^*	35.8*^a^*	0.91*^a^*	55.5	36	32*^a^*	31.1	30.2*^a^*
		IP chemotherapy	45 Gy/1.5 Gy/30f, BID	4 cycles: irinotecan, cisplatin	129			33.7*^a^*		51.7	36.2	30.8*^a^*	28.5	27.2*^a^*
Faivre-Finn, (25)	2008.07–2013.11	Once-daily RT	66 Gy/2 Gy/33f, QD	4~6 cycles: cisplatin, etoposide	270	3.8	0.85*^a^*	–	0.89*^a^*	NA	NA	NA	NA	NA
		Twice-daily RT	45 Gy/1.5 Gy/30f, BID	4~6 cycles: cisplatin, etoposide	273			–		NA	NA	NA	NA	NA
Bogart, (5)	2008.03–2019.12	Once-daily RT	70 Gy/2 Gy/35f, QD	4 cycles: etoposide, cisplatin or etoposide carboplatin	325	2.8	0.94*^a^*	34*^a^*	0.96*^a^*	54.4	36*^a^*	31.4	27.6	24*^a^*
		Twice-daily RT	45 Gy/1.5 Gy/30f, BID	4 cycles: etoposide, cisplatin or etoposide carboplatin	313			29*^a^*		54.4	36*^a^*	29.4	27.6	25*^a^*

^a^Data directly reported in the text.

^b^Data for time to progression.

CRT, chemoradiotherapy; EP, etoposide plus cisplatin; IP, irinotecan plus cisplatin; NA, not available; OS, overall survival; PFS, progression-free survival; RT, radiotherapy; TTP, time to progression.

**Table 2:** Summary of 22 phase II and retrospective studies in the current meta-analysis.

Study	Study period	Treatment arm	Radiotherapy dose	Chemotherapy regimen	No. of patients	Median follow-up, year	OS, %	PFS/TTP, %			
							5-year	2-year	3-year	4-year	5-year
*Phase II randomized controlled trial (n = 4)*											
Grønberg, (27)	2005.05–2011.01	Once-daily RT	42 Gy/2.8 Gy/15f, QD	4 cycles: etoposide, cisplatin or etoposide carboplatin	84	4.9	25	26*^a^*	26	23.1	23.1
		Twice-daily RT	45 Gy/1.5 Gy/30f, BID	3 cycles: etoposide, cisplatin or etoposide carboplatin	73		23.3	29*^a^*	20.9	17.3	17.3
Grønberg, (28)	2014.07–2018.06	Standard-dose RT	45 Gy/1.5 Gy/30f, BID	4 cycles: etoposide, cisplatin	81	NA	37.8	45.2	37.6	34.1	30.4
		High-dose RT	60 Gy/1.5 Gy/40f, BID	4 cycles: etoposide, cisplatin	89		29	33.2	30.2	28.7	26
Peters, (29)	2015.12–2019.04	observation	56 Gy/2 Gy/28f, QD or 45 Gy/1.5 Gy/30f, BID	4 cycles: etoposide, cisplatin or etoposide carboplatin	75	1.9	35.5	40.3*^a^*	40.3*^a^*	40.3*^a^*	NA
		consolidation immunotherapy	56 Gy/2 Gy/28f, QD or 45 Gy/1.5 Gy/30f, BID	4 cycles: etoposide, cisplatin or etoposide carboplatin	78		51*^a^*	43.2*^a^*	43.2*^a^*	43.2*^a^*	NA
Qiu, (30)	2015.01–2019.06	Once-daily RT	65 Gy/2.5 Gy/26f, QD	4-6 cycles: etoposide, cisplatin	88	2.0	44.7	42.3*^a^*	37.2*^a^*	37.2	37.2
		Twice-daily RT	45 Gy/1.5 Gy/40f, BID	4-6 cycles: etoposide, cisplatin	94		27.7	28.4*^a^*	19.9*^a^*	19.9	19.9
*Single-arm phase II study (n = 8)*											
Hügli, (31)	1993.07–1998.05		45 Gy/1.5 Gy/30f, BID	6 cycles: etoposide, cisplatin	52	3.8	32*^a^*	32.3	30*^a^*	26	26
Thomas, (32)	1985.04–1986.05		45 Gy/1.8 Gy/25f, QD	1st, 2nd, 3rd cycle: cisplatin, etoposide, vincristine. 4th, 5th cycle: methotrexate, vincristine, etoposide, doxorubicin, cyclophosphamide	114	6.5	26.1	33.4	28.1	26.4	23.6
Ettinger, (33)	1996.11–1998.03		45 Gy/1.5 Gy/30f, BID	4 cycles: etoposide, cisplatin, paclitaxel	53	NA	22.3	27.8	25.4	23.8	22
Yilmaz, (34)	2001.02–2007.03		50~60 Gy/2 Gy/25~30f, QD	6 cycles: etoposide, carboplatin	47	1.1	7	10	10	7	7
CALGB 39808, (35)	1999.03–2000.06		70 Gy/2 Gy/35f, QD	1st, 2nd cycle: topotecan, paclitaxel, 3rd, 4th, 5th cycle: etoposide, carboplatin	62	6.5	19*^a^*	29*^a^*	27.3	22.7	21.1
CALGB 30002, (36)	2001.06–2003.01		70 Gy/2 Gy/35f, QD	1st, 2nd cycle: etoposide, topotecan, paclitaxel, 3rd, 4th, 5th cycle: etoposide, carboplatin	63		23*^a^*	25*^a^*	25	25	23.5
CALGB 30206, (37)	2003.11–2005.09		70 Gy/2 Gy/35f, QD	1st, 2nd cycle: cisplatin, irinotecan, 3rd, 4th, 5th cycle: etoposide, carboplatin	75		17*^a^*	21*^a^*	21	15.8	14.4
Xia, (38)	2007.07–2012.02		55 Gy/2.5 Gy/22f, QD	4~6 cycles: etoposide, cisplatin	59	1.6	34.3	49*^a^*	43.9	37.1	37.1
*Retrospective study (n=10)*											
Kamath, (39)	1986.07–1994.08		30–50 Gy	Etoposide, cisplatin or etoposide carboplatin	34	2.4	32*^a^*	35*^a^*	31*^a^*	31*^a^*	31*^a^*
Khanfir, (40)	1997.12–2006.1		Meidan:60 Gy	Platinum-based chemotherapy	69	3.0	18.4	32.9	23*^a^*	16.6	16.6
Han, (41)	2004.07–2009.07	Involved-field irradiation	60 Gy/2 Gy/30f, QD or 45 Gy/1.5 Gy/30f, BID	Platinum-based doublets	50	2.8	23.4	34.5	24.2	24.2	24.2
		Elective nodal irradiation	60 Gy/2 Gy/30f, QD or 45 Gy/1.5 Gy/30f, BID	Platinum-based doublets	30		49.8	46.7	42.8	42.8	42.8
Wang, (42)	2009.01–2011.12	Early RT	50~66 Gy/1.8~2.1 Gy/f, QD	2~6 cycles: platinum-based doublets	89	3.7	35.9*^b^*	39.5*^b^*	37.9*^b^*	35.5*^b^*	35.5*^b^*
		Late RT	50~66 Gy/1.8~2.1 Gy/f, QD	2~6 cycles: platinum-based doublets	57		14.6*^b^*	25.8*^b^*	18.9*^b^*	18.9*^b^*	18.9*^b^*
Morimoto, (43)	2004.01–2013.10		45 Gy/1.5 Gy/30f, BID	4 cycles: etoposide, cisplatin or etoposide carboplatin	81	1.8	26.2*^a^*	28*^a^*	24.5*^a^*	24.5	19*^a^*
Zhang, (44)	2010.01–2013.12	Conventionally fractionated RT	≥56 Gy/2 Gy/≥28 Gy, QD	4~6 cycles: etoposide, cisplatin or etoposide carboplatin	101	2.5	25.6	32.4*^a^*	23.2	22.7	22.7
		Hyperfractionated RT	55 Gy/2.5 Gy/22f, QD	4~6 cycles: etoposide, cisplatin or etoposide carboplatin	69		21.3	33.5*^a^*	29.7	29.7	24.8
Jeong, (45)	2005.08–2014.03		≥45 Gy	4~6 cycles: etoposide, cisplatin	101	2.2	26.7*^a^*	33.9	29.5*^a^*	28.3	28.3*^a^*
Zayed, (46)	2000–2013	Conventionally fractionated RT	≥58 Gy/2 Gy/≥29f, QD	NA	61	5.0	24*^a^*	30.6	25	19.2	19.2
		Hyperfractionated RT	37~50 Gy/≥2.1 Gy/f, QD	NA	56		26.2*^a^*	35.9	30.2	26.2	21.9
Atci, (47)	2002–2019		45 Gy/1.5 Gy/30f, BID	etoposide, cisplatin or etoposide carboplatin	89	1.7	34.3*^a,b^*	41.9*^b^*	27.7*^a,b^*	26.4*^b^*	24.9*^a,b^*
Doshita, (48)	2002.09–2018.02		45 Gy/1.5 Gy/30f, BID	etoposide, cisplatin or etoposide carboplatin	120	6.0	41.8*^a^*	41.2	37.6*^a^*	35.6	33.6*^a^*

^a^Data directly reported in the text.

^b^Data for time to progression.

NA, not available; OS, overall survival; PFS, progression-free survival; RT, radiotherapy; TTP, time to progression.

### Endpoint Definition

OS was defined as the time from randomization, registration, diagnosis or the first day of treatment to death. PFS was defined as the time from randomization, registration, diagnosis or the first day of treatment to disease progression or death. TTP was defined as the time from randomization, registration, diagnosis or the first day of treatment to disease progression (**Supplementary Tables 2**, **3**). As surrogate endpoints were defined differently between the trials, two investigators (YY and JY,W) labelled an endpoint of a trial as PFS or TTP according to our established definitions. For the literature without detailed definition of PFS/TTP, we tried to contact authors of original research, otherwise the definition from the text was adopted.

### Quality Assessment

The quality of the candidate Phase II, III RCTs was evaluated on 7 domains according to the Cochrane Collaboration tool. The trials were excluded if high risk of bias in any domain was detected (**Supplementary Table 4**).

The quality of the candidate single-arm phase II, and retrospective studies was assessed in 3 domains with 9 items according to Newcastle-Ottawa Scale for cohort study. The studies were excluded if their scores were less than 6 points (**Supplementary Table 5**).

### Statistical Analysis

#### Correlation Evaluation

The correlations between surrogate endpoints and OS in phase III RCTs were performed at both trial- and arm-level. At trial level, the correlation of HRs for PFS/TTP and HRs for OS was qualified through a linear regression model, weighted by trial size. At arm-level, the linear correlation between the 1-, 2-, 3-, 4-, and 5-year PFS/TTP rates and 5- year OS rate was also evaluated by the linear regression model, with weight equal to each treatment-arm sample size. The coefficient of determination R^2^ was calculated to assess the strength of correlation. R^2^ values of 0–0.25, 0.25–0.5, 0.5–0.75, 0.75–0.9, 0.9–1 indicated poor, moderate, good, very good and excellent correlation. If the R^2^ value was greater than 0.75, the following sensitivity analysis, leave-one-out cross-validation, and external validation were performed. If R^2^ values showed great discrepancy between two adjacent time points, further subdivision of the time period and corresponding PFS/TTP rate extraction was performed to find a cut-off value.

#### Sensitivity Analysis

Phase III RCTs were classified into four subgroups depending on study designs (**Supplementary Table 6**). To assess the consistency and robustness of prediction models across different settings, sensitivity analyses were performed by leaving each subgroup of trials out at a time. The coefficient of determination R^2^ value and its 95% CI were calculated by the weighted linear regression method mentioned above.

#### Leave-One-Out Cross-Validation

To assess the accuracy of prediction models, a leave-one-out cross-validation approach was performed. Each trial or treatment arm was left out once, and at each leave-one-out step a linear regression model was rebuilt on the other trials or arms (n-1). This model was then applied to the left-out trial or arm and the corresponding 95% prediction interval was calculated to compare the predicted and actually observed treatment effect on OS.

#### External Validation of Phase III RCT Prediction Model

The arm-level predictive linear regression models established by phase III RCTs were applied to the phase II and retrospective studies for external validation. The predicted 5-year OS rate was calculated from the actual 1–5-year PFS/TTP rates in the phase II or retrospective studies using the established linear regression model from the phase III RCTs. More specifically, the equation “5-year OS = α × 1-, 2-, 3-, 4-, or 5-year PFS/TTP + β” was derived from the phase III RCTs. The reported 1~5-year PFS/TTP rates derived from the phase II and retrospective studies were put into the equation, then the predicted 5-year OS rate was generated. The actual and predicted 5-year OS rates were plotted in scatter plots.

Statistical analysis was performed with SPSS (version 26.0), data visualization was performed using the ggplot2 package in R software (version 4.0.4) and GraphPad Prism (version 8.4.0).

## Results

### Study Characteristics

A total of 4,212 records were searched, and 40 records were screened to quality assessment. Among the 40 records, 3 records (40, 49, 50) were excluded for high risk of bias and 37 records, consisting of 15 phase III RCTs (4, 5, 13–25), 12 phase II (27–38), and 10 retrospective studies (39, 41–48, 51), were finally included for analysis (**Supplementary Figure 1**). Long-term survival data of three single-arm phase II studies (35–37) were updated in another report (52). The HRs for PFS and OS of a phase III trial (23) were corrected later (53). Thus, we conducted meta-analysis with these updated data.

### Trial-Level Correlation Between PFS/TTP on OS in Phase III RCTs

**Figure 1:**
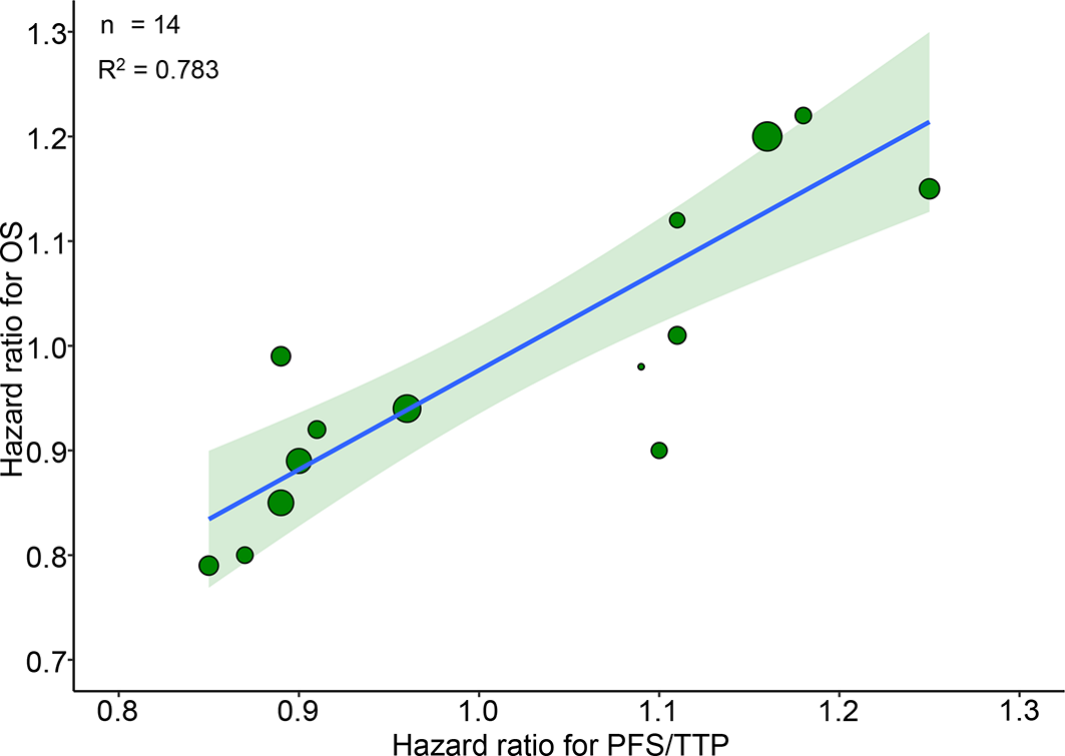
Trial-level correlation between hazard ratios for OS and PFS/TTP in phase III RCTs. Green circles represent trials with a size proportional to the number of patients, blue line for the estimated regression line and the light green zone for 95% confidence intervals. OS, overall survival; PFS/TTP, progression free survival/time to progression; RCTs, randomized controlled trials.

**Figure 2:**
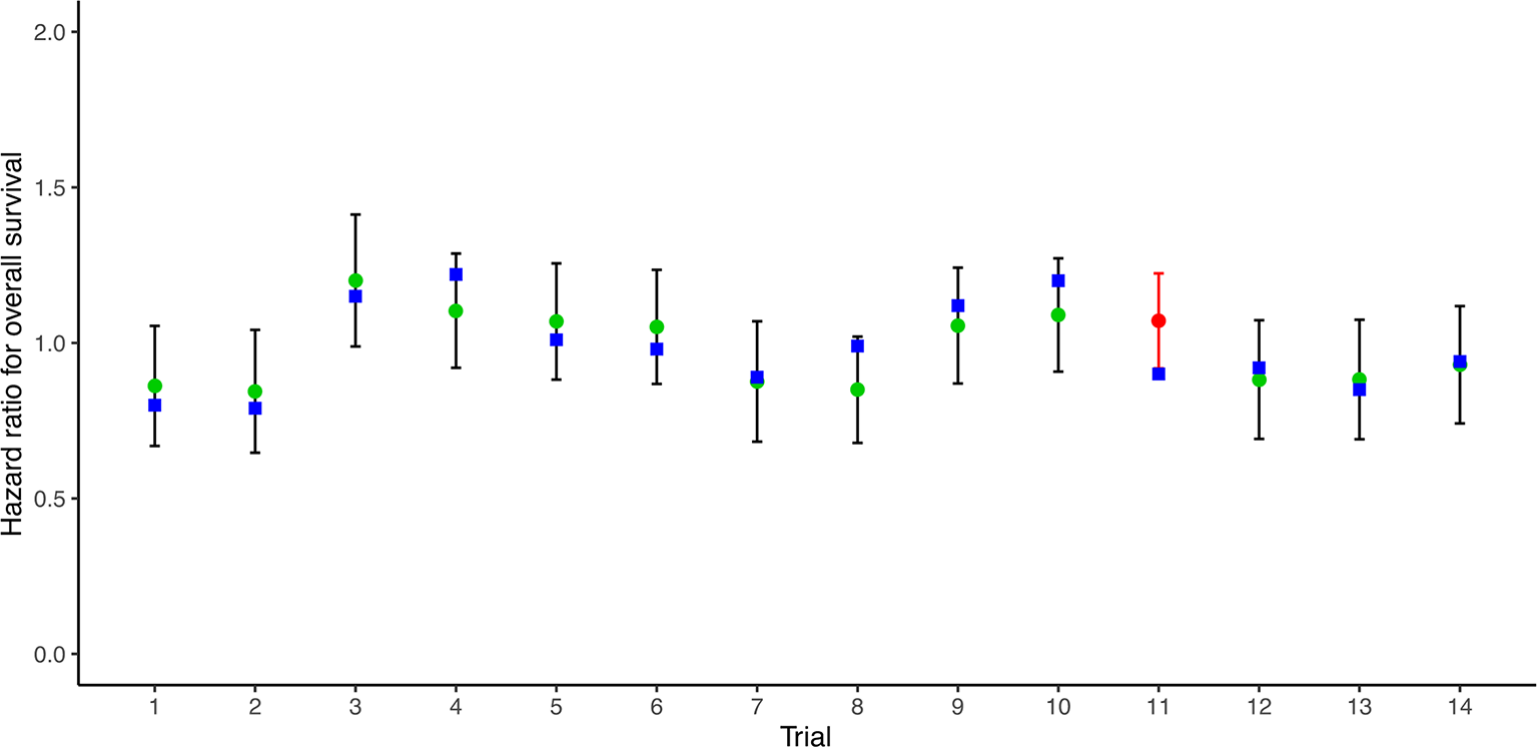
Leave-one-out cross-validation analysis of the prediction of HR for OS based on HR for PFS/TTP. Green circles represent predicted hazard ratio for OS, vertical lines for 95% prediction intervals, and blue squares for observed hazard ratios for OS. Red circles and lines indicate that the observed HR is beyond the 95% prediction intervals. HR, hazard ratio; OS, overall survival; PFS/TTP, progression-free survival/time to progression.

14 RCTs reported pairs of HRs for PFS/TTP and OS. A very good correlation was observed between 14 pairs of HRs for PFS/TTP and OS (R^2^ = 0.783, 95% CI 0.771–0.794) (**Figure 1**). Sensitivity analysis showed very good correlations and robust consistency in most subgroups, except when leaving out 6 trials of different radiotherapy model (R^2^ = 0.645, 95% CI 0.587–0.674) (**Supplementary Figure 2A**). This result was expected as the subgroup of different radiotherapy model close to half of the number of trials. Exclusion of these trials probably results in a lower correlation and wider confidence interval. The cross-validation showed good consistency, as the observed HRs for OS were all in the 95% prediction intervals in 13 of 14 trials, and the HRs were very close to 95% prediction intervals in the remaining one trial (23) (**Figure 2**).

### Treatment Arm-Level Correlation Between PFS/TTP and OS in Phase III RCTs

**Figure 3:**
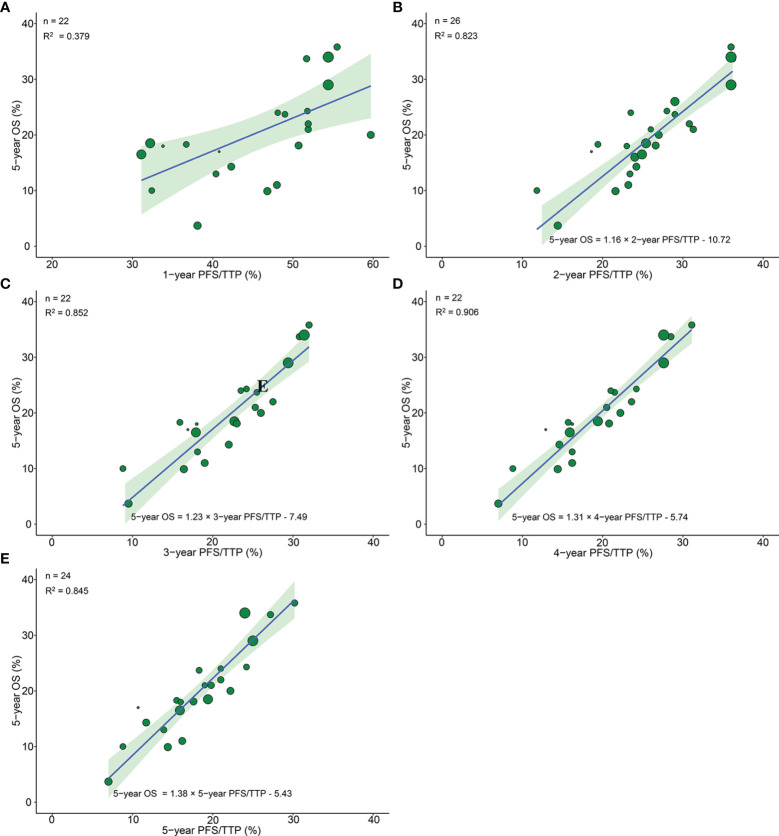
Treatment arm-level correlation between 5-year OS and 1-year PFS/TTP **(A)**, 2-year PFS/TTP **(B)**, 3-year PFS/TTP **(C)**, 4-year PFS/TTP **(D)**, 5-year PFS/TTP **(E)** in phase III RCTs. Green circles represent treatment arms with a size proportional to the number of patients, blue lines for the estimated regression lines and the light green zones for 95% confidence intervals. OS, overall survival; PFS/TTP, progression free survival/time to progression; RCTs, randomized controlled trials.

26 arms from 13 phase III RCTs reported 5-year OS, among which 22 arms from 11 trials, 26 arms from 13 trials, 22 arms from 11 trials, 22 arms from 11 trials, and 24 arms from 12 trials reported 1-, 2-, 3-, 4-, and 5-year PFS/TTP, respectively. The correlation between 1-year PFS/TTP and 5-year OS was moderate (R^2^ = 0.379, 95% CI 0.358–0.394). However, very good correlations were observed analyzing 26 pairs of 2-year PFS/TTP and 5-year OS (R^2^ = 0.823, 95% CI 0.814–0.832), 22 pairs of 3-year PFS/TTP and 5-year OS (R^2^ = 0.852, 95% CI 0.843–0.859), and 22 pairs of 5-year PFS/TTP and 5-year OS (R^2^ = 0.845, 95% CI 0.834–0.852). Moreover, an excellent correlation was observed analyzing 26 pairs of 4-year PFS/TTP and 5-year OS (R^2^ = 0.906, 95% CI 0.901–0.910) (**Figure 3**).

Because R^2^ showed great discrepancy between 1-year PFS/TTP and 2-year PFS/TTP, we further divided the time duration from 1 to 2 years into 5 parts with 4 time points (1.2, 1.4. 1.6, and 1.8 years); corresponding PFS/TTP rates were extracted to calculate R^2^ with a 5-year OS rate. The plot of R^2^ and PFS/TTP time showed that the best cutoff time point was 2 years, which indicated that the ≥2-year PFS/TTP rate was the valid surrogate endpoint (**Supplementary Figure 3**).

Sensitivity analysis showed very good correlations and robust consistency in most subgroups, except when leaving out subgroups of different radiotherapy model due to fewer trials (**Supplementary Figures 2B–E**).

**Figure 4:**
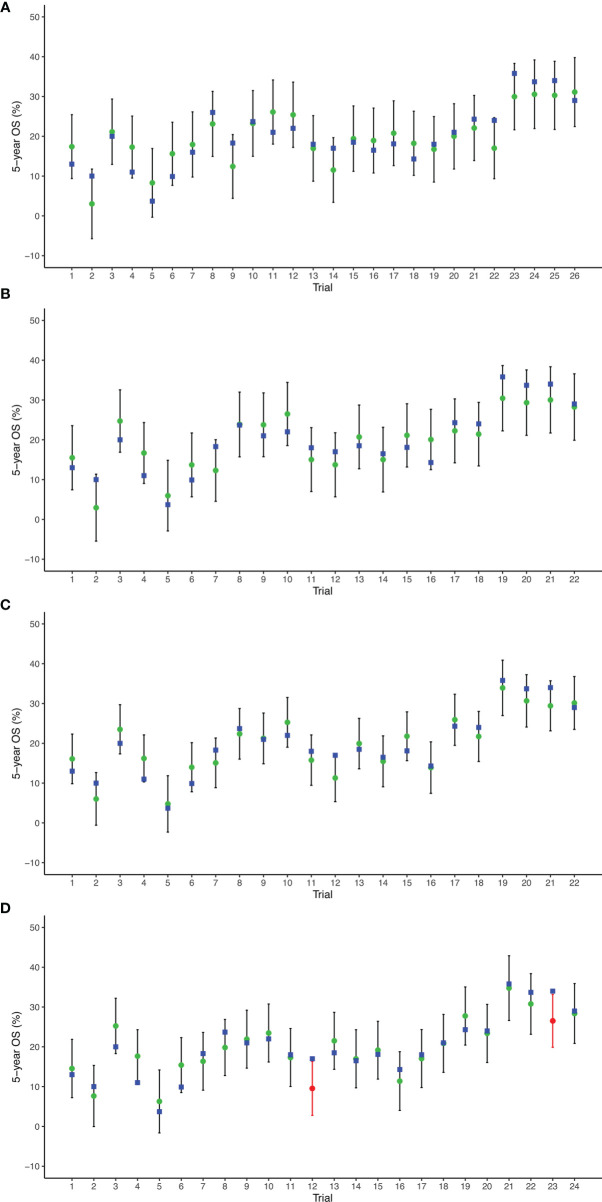
Leave-one-out cross-validation analysis of the prediction of 5-year OS based on 2-year PFS/TTP **(A)**, 3-year PFS/TTP **(B)**, 4-year PFS/TTP **(C)**, and 5-year PFS/TTP **(D)**. Green circles represent predicted 5-year OS, vertical lines for 95% prediction intervals, and blue squares for observed 5-year OS. Red circles and lines indicate that observed 5-year OS is beyond the 95% prediction intervals. OS, overall survival; PFS/TTP, progression-free survival/time to progression.

The prediction results of cross-validation analyses showed that the observed 5-year OS rate fell within the 95% prediction intervals in all arms based on 2-, 3-, and 4-year PFS/TTP. With respect to the 5-year PFS/TTP, the observed 5-year OS rates were all in the 95% prediction intervals in 22 of 24 arms, and the 5-year OS rates of the remaining two trials (5, 18) are very close to the 95% prediction intervals (**Figure 4**).

These findings indicated that improvements in 2–5-year PFS/TTP are strongly associated with a higher 5-year OS.

### External Validation of the Correlation Between PFS/TTP and OS

**Figure 5:**
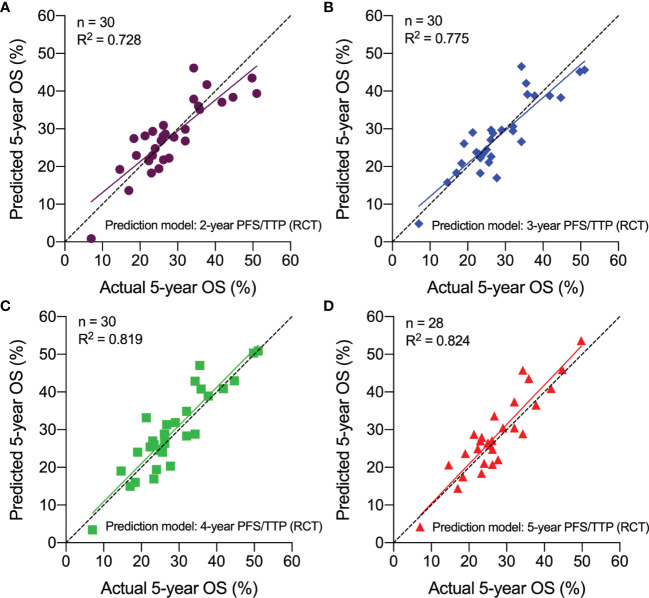
External validation of the correlation between PFS/TTP and OS in Phase II and retrospective studies. The predicted 5-year OS based on actual 2-year PFS/TTP **(A)**, 3-year PFS/TTP **(B)**, 4-year PFS/TTP **(C)**, and 5-year PFS/TTP **(D)** is plotted against the actual 5-year OS. OS, overall survival; PFS/TTP, progression-free survival/time to progression; RCT, randomized controlled trial.

30 treatment arms from 12 phase II and 10 retrospective studies were used for external validation. Using the arm-level prediction models from the phase III RCTs, we calculated the predicted 5-year OS rate for each phase II and retrospective studies using the actual 2-, 3-, 4-, or 5-year PFS/TTP rate. The actual and predicted 5-year OS rates were plotted in scatter plots, which indicated that the predicted 5-year OS was approximated to the actual 5-year OS. The predicted 5-year OS rate greatly correlated with the actual 5-year OS rate, with the R^2^ ranging from 0.728 to 0.824 (**Figure 5**). These results validated the hypothesis that PFS/TTP is the efficient surrogate endpoint of OS.

## Discussion

This is the first study combining data from high-quality Phase III RCTs, Phase II studies, and retrospective studies to explore the efficacy of PFS or TTP as a surrogate endpoint of OS in patients with limited-stage SCLC who underwent chemoradiotherapy. Previous meta-analyses have assessed surrogate endpoints in other types or stages of lung cancer. Mauguen et al. (11) indicated that PFS and DFS are valid surrogate endpoints in locally advanced non-small-cell lung cancer. As for extensive-stage SCLC, Foster et al. (12) firstly reported that PFS was a potential surrogate endpoint using individual data from 6 single-arm and 3 RCTs in 2011 and then validated the results by seven new phase II/III trials in 2015 (54). However, analysis of surrogate endpoints in limited-stage SCLC has never been tested.

Our study showed that there were strong correlations between PFS/TTP and OS at the trial level, and 2–5-year PFS/TTP and 5-year OS at the treatment arm level. The coefficient R^2^ ranged from 0.783 to 0.907, which indicated that nearly 78.3%–90.7% of the variation on OS can be indicated by PFS or TTP. The sensitivity analysis showed good consistency across different settings, and the cross-validation also showed good accuracy of the prediction models. The external validation with phase II and retrospective studies showed excellent agreement between the actual and predicted 5-year OS rates derived from the established linear regression models. These findings confirmed the feasibility of taking PFS/TTP as the primary endpoint for clinical trials of LS-SCLC.

However, the predictive value of 1-year PFS/TTP for 5-year OS was quite lower compared with that of the 2-year PFS/TTP (correlation R^2^ 0.379 vs. 0.823), which indicated that 1-year PFS/TTP was not an appropriate surrogate endpoint. PFS/TTP data of phase III RCT (**Table 1**) showed that around 50% of patients without progression relapsed during the second year after upfront treatment. From the third year, the PFS/TTP did not reduce significantly. **Table 2** also showed that the PFS/TTP was relatively stable after 2 years of chemoradiotherapy in phase II and retrospective studies. This decreasing trend of PFS/TTP in LS-SCLC was consistent with our clinical experience. Thus, longer follow-up time such as 2–5-year PFS/TTP was necessary.

There has been debate on how a surrogate endpoint should be considered as valid. We employed the correlation approach which has been used to assess the possibility of PFS or TTP as a surrogate endpoint for OS in locally advanced NSCLC (11), nasopharyngeal carcinoma (8), and diffuse large B-cell lymphoma (10). Candidate surrogate endpoints could be valid only if the correlation coefficient was greater than 0.75 (55).

Exploring effective treatment or drugs for SCLC is urgent as its prognosis is still poor compared with other malignancy. There was no improvement of outcome for extensive-stage SCLC in the past more than three decades until atezolizumab was added in the classic regimen of etoposide and cisplatin as first-line chemotherapy (56). However, the immunotherapy plus chemotherapy only prolonged the median OS by 2 months compared with chemotherapy alone after more than a 2-year study period. For locally advanced lung cancer, new treatments have significantly increased the OS in NSCLC based on results of the PACIFIC trial (57), and the mature OS was achieved after 6 years from the beginning of the first patients enrolled (58). For limited-stage SCLC under standard concurrent chemoradiotherapy followed by prophylactic cranial irradiation, 5-year OS was still low ranging from 26% to 34% and did not change in the past two decades (4, 25). There are several ongoing phase II and III trials investigating the PD-1/PD-L1 consolidation immunotherapy (59–62). High-level evidence for immunotherapy as concurrent of consolidation treatment has not been reported until now. However, parts of these ongoing trials (59, 60) have already defined PFS as the primary endpoint, OS as the second endpoint. Given that no study has reported valid surrogate endpoints for limited-stage SCLC, our analysis is of great importance to provide a rationale to define PFS or TTP as the primary endpoint in clinical trials, so as to speed up introducing novel effective agents to improve outcome of LS-SCLC.

Another advantage of this study is comprehensively enrolled published literatures with high quality and proper sample size. In addition to the strong correlations demonstrated by phase III RCTs, the positive relationships between 2–5-year PFS/TTP and 5-year OS rates were externally validated by independent data from phase II and retrospective studies. The validation method was unique and firstly used in diffuse large B-cell lymphoma (10) by our department, which showed good efficacy to find early surrogate endpoints. This time, the validation method was used again in limited-stage SCLC to improve the reliability of the conclusions. Moreover, our study found multiple time points between 1-year and 2-year PFS/TTP which were not suitable, indicating that the 2-year PFS/TTP rate or more was really valid when used as a primary endpoint.

One major statistical challenge is inconsistencies and absences of the definition of endpoints across the trials in the current study. The starting time for endpoints was defined from randomization, registration, diagnosis, or the first day of treatment, differently. As SCLC is one of the most aggressive cancers, a difference of 1 or 2 months caused by definition of starting time may result in bias from different arms. Second, this is a literature-based systematic review and meta-analysis without individual patient data; therefore, a potential publication bias cannot be excluded. Third, the prediction models were based on data of patients who received first-line combined chemoradiotherapy, so the extrapolation to other treatments was cautious, especially when more effective second or more line treatments were developed in the future. Nevertheless, LS-SCLC patients usually died after disease progression because there are still no effective second-line treatments nowadays (25).

In conclusion, the current study provides first literature-based evidence to evaluate the correlation of PFS/TTP with OS in patients with limited-stage SCLC. The finding supports PFS/TTP as a valid surrogate endpoint for OS in LS-SCLC patients who underwent combined upfront chemoradiotherapy.

## Data Availability Statement

The original contributions presented in the study are included in the article/**Supplementary Material**. Further inquiries can be directed to the corresponding author.

## Author Contributions

Concept and design: YL, LW, NB. Acquisition, analysis, or interpretation of data: YY, JW, LW, NB. Drafting of the manuscript: YY, JW. Critical revision of the manuscript for important intellectual content: all authors. Statistical analysis: YY, TZ, YL. Obtained funding: LW, NB. All authors contributed to the article and approved the submitted version.

## Funding

This study is supported by the National Natural Science Foundation of China (81572971) and Beijing Hope Run Special Fund of Cancer Foundation of China (LC2018A04).

## Conflict of Interest

The authors declare that the research was conducted in the absence of any commercial or financial relationships that could be construed as a potential conflict of interest.

## Publisher’s Note

All claims expressed in this article are solely those of the authors and do not necessarily represent those of their affiliated organizations, or those of the publisher, the editors and the reviewers. Any product that may be evaluated in this article, or claim that may be made by its manufacturer, is not guaranteed or endorsed by the publisher.
