# Corrigendum: Progression-free survival and time to progression as potential surrogate endpoints for overall survival in chemoradiotherapy trials in limited-stage small-cell lung cancer: A systematic review and meta-analysis

**DOI:** 10.3389/fonc.2022.1007862

**Published:** 2022-08-24

**Authors:** Yin Yang, Jianyang Wang, Wenqing Wang, Tao Zhang, Jingjing Zhao, Yu Wang, Yexiong Li, Luhua Wang, Nan Bi

**Affiliations:** ^1^ Department of Radiation Oncology, National Cancer Center/National Clinical Research Center for Cancer/Cancer Hospital, Chinese Academy of Medical Sciences and Peking Union Medical College, Beijing, China; ^2^ Department of Radiation Oncology, National Cancer Center/National Clinical Research Center for Cancer/Cancer Hospital & Shenzhen Hospital, Chinese Academy of Medical Sciences and Peking Union Medical College, Shenzhen, China

**Keywords:** Limited-stage small-cell lung cancer, surrogate endpoint, overall survival, progression-free survival, time to progression, chemoradiotherapy

In the published article, there was an error in the legend for **Figure 1** and **Figure 3** as published, regarding the light green zone for 95% prediction intervals. The corrected legends appear below.

**Figure 1:**
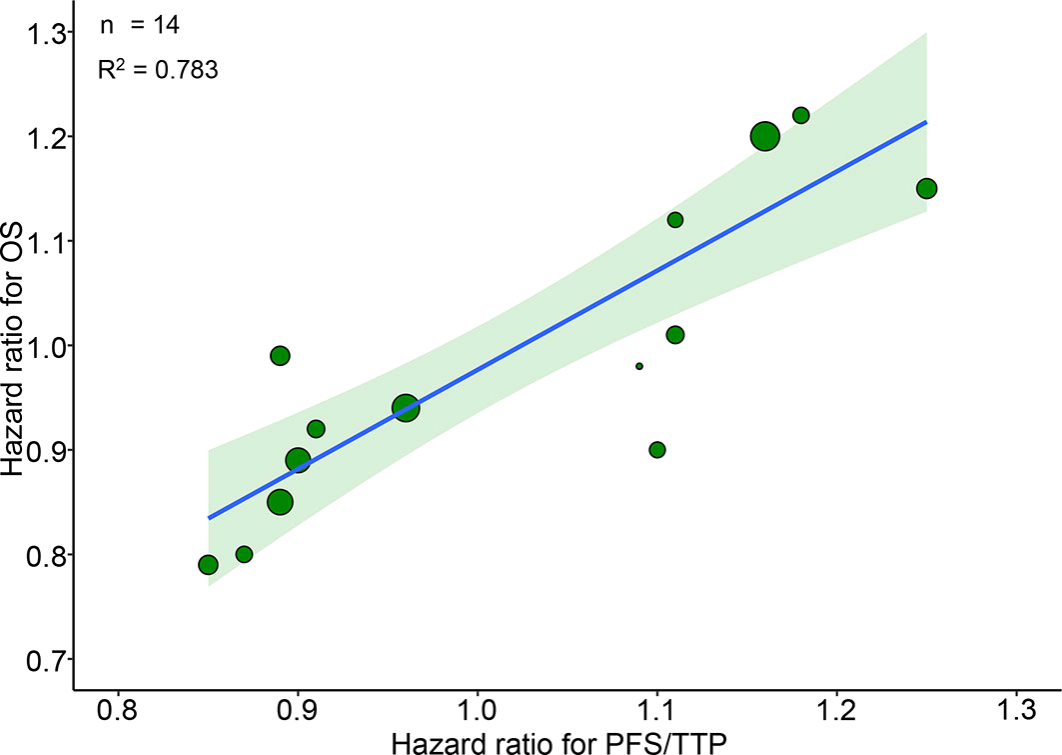
Trial-level correlation between hazard ratios for OS and PFS/TTP in phase III RCTs. Green circles represent trials with a size proportional to the number of patients, blue line for the estimated regression line and the light green zone for 95% confidence intervals. OS, overall survival; PFS/TTP, progression free survival/time to progression; RCTs, randomized controlled trials.

**Figure 3:**
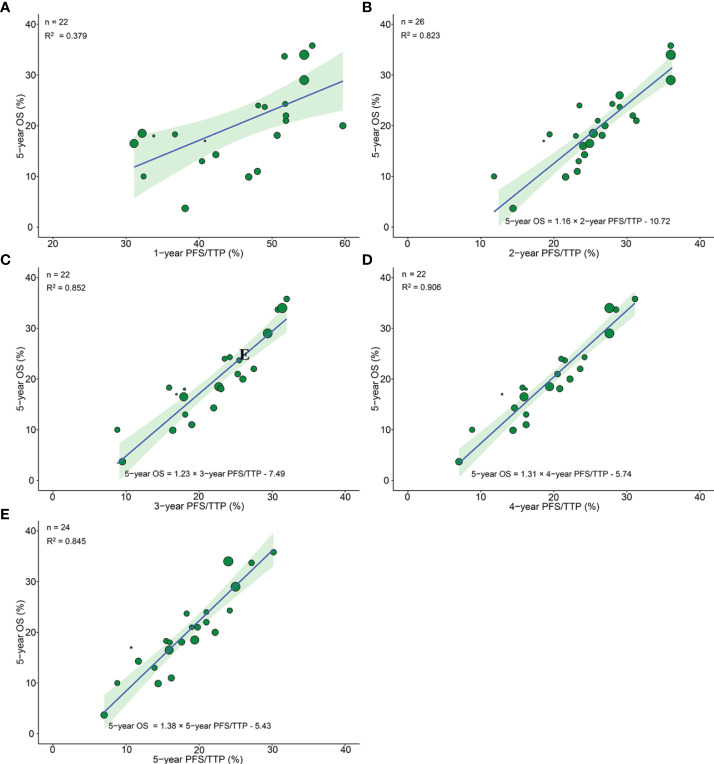
Treatment arm-level correlation between 5-year OS and 1-year PFS/TTP **(A)**, 2-year PFS/TTP **(B)**, 3-year PFS/TTP **(C)**, 4-year PFS/TTP **(D)**, 5-year PFS/TTP **(E)** in phase III RCTs. Green circles represent treatment arms with a size proportional to the number of patients, blue lines for the estimated regression lines and the light green zones for 95% confidence intervals. OS, overall survival; PFS/TTP, progression free survival/time to progression; RCTs, randomized controlled trials.

The authors apologize for this error and state that this does not change the scientific conclusions of the article in any way. The original article has been updated.

## Publisher’s note

All claims expressed in this article are solely those of the authors and do not necessarily represent those of their affiliated organizations, or those of the publisher, the editors and the reviewers. Any product that may be evaluated in this article, or claim that may be made by its manufacturer, is not guaranteed or endorsed by the publisher.

