# Supplementary material for: Progression-Free Survival and Time to Progression as Potential Surrogate Endpoints for Overall Survival in Chemoradiotherapy Trials in Limited-Stage Small-Cell Lung Cancer: A Systematic Review and Meta-Analysis
Source: Front Oncol. 2022 Jan 28;12:810580. doi: 10.3389/fonc.2022.810580 (PMC8834538; doi:10.3389/fonc.2022.810580)
Supplement: Supplementary file 1 [file DataSheet_1.docx]

**Supplementary Table 1. Queries used for systematic search.**

**Supplementary Table 2. The definition of PFS and TTP in phase Ⅲ randomized controlled trials.**

**Supplementary Table 3. The definition of PFS and TTP in phase Ⅱ and retrospective studies.**

**Supplementary Table 4. The assessment of risk of bias in phase Ⅲ, Ⅱ randomized controlled trials by the Cochrane Collaboration tool.**

**Supplementary Table 5. The assessment of risk of bias in single-arm phase Ⅱ, and retrospective studies by the Newcastle – Ottawa quality assessment scale for cohort study.**

**Supplementary Table 6. Four subgroups for phase Ⅲ randomized controlled trials.**

**Supplementary Figure 1. Flow diagram of research selection.**

**Supplementary Figure 2. Sensitivity analysis across different settings.**

**Supplementary Figure 3. Plot of R^2^ and PFS/TTP year.**

# Supplementary Table 1. Queries used for systematic search.

| Sources | Queries |
| --- | --- |
| PubMed | (“Limited”[Title/Abstract]) AND (“Small Cell Lung Carcinoma”[MeSH Terms] OR “Small Cell Lung Cancer”[Title/Abstract] OR “Oat Cell Lung Cancer”[Title/Abstract] OR “Small Cell Cancer Of The Lung”[Title/Abstract] OR “Carcinoma, Small Cell Lung”[Title/Abstract] OR “Oat Cell Carcinoma of Lung”[Title/Abstract]) AND (“Radiotherapy”[MeSH Terms] OR “Radiotherapies”[Title/Abstract]) OR “Radiation Therapy”[Title/Abstract] OR “Radiation Therapies”[Title/Abstract] OR “Therapies, Radiation”[Title/Abstract] OR “Therapy, Radiation”[Title/Abstract] OR “Radiation Treatment”[Title/Abstract] OR “Radiation Treatments”[Title/Abstract] OR “Treatment, Radiation”[Title/Abstract] OR “Radiotherapy, Targeted”[Title/Abstract] OR “Radiotherapies, Targeted”[Title/Abstract] OR “Targeted Radiotherapies”[Title/Abstract] OR “Targeted Radiotherapy”[Title/Abstract] OR “Targeted Radiation Therapy”[Title/Abstract] OR “Radiation Therapies, Targeted”[Title/Abstract] OR “Targeted Radiation Therapies”[Title/Abstract] OR “Therapies, Targeted Radiation”[Title/Abstract] OR “Therapy, Targeted Radiation”[Title/Abstract] OR “Radiation Therapy, Targeted”[Title/Abstract] OR “Chemoradiotherapy”[Title/Abstract] OR “Chemoradiotherapies”[Title/Abstract] OR “Radiochemotherapy”[Title/Abstract] OR “Radiochemotherapies”[Title/Abstract]) |
| Embase | ('limited':ab,ti)AND ('small cell lung cancer'/exp OR'small cell lung carcinoma':ab,ti OR 'oat cell lung cancer':ab,ti OR 'small cell cancer of the lung':ab,ti OR 'carcinoma, small cell lung':ab,ti OR 'oat cell carcinoma of lung':ab,ti) AND('radiotherapy'/exp OR 'radiotherapies':ab,ti OR 'radiation therapy':ab,ti OR 'radiation therapies':ab,ti OR 'therapies, radiation':ab,ti OR 'therapy, radiation':ab,ti OR 'radiation treatment':ab,ti OR 'radiation treatments':ab,ti OR 'treatment, radiation':ab,ti OR 'radiotherapy, targeted':ab,ti OR 'radiotherapies, targeted':ab,ti OR 'targeted radiotherapies':ab,ti OR 'targeted radiotherapy':ab,ti OR 'targeted radiation therapy':ab,ti OR 'radiation therapies, targeted':ab,ti OR 'targeted radiation therapies':ab,ti OR 'therapies, targeted radiation':ab,ti OR 'therapy, targeted radiation':ab,ti OR 'radiation therapy, targeted':ab,ti OR 'chemoradiotherapy':ab,ti OR 'chemoradiotherapies':ab,ti OR 'radiochemotherapy':ab,ti OR 'radiochemotherapy':ab,ti OR 'radiochemotherapies':ab,ti) |
| The Cochrane Library | (“Limited”:ti,ab,kw) AND (“Small Cell Lung Carcinoma”[MeSH Terms] OR “Small Cell Lung Cancer”:ti,ab,kw OR “Oat Cell Lung Cancer”:ti,ab,kw OR “Small Cell Cancer Of The Lung”:ti,ab,kw OR “Carcinoma, Small Cell Lung”:ti,ab,kw OR “Oat Cell Carcinoma of Lung”:ti,ab,kw) AND(“Radiotherapy”[MeSH Terms] OR “Radiotherapies”:ti,ab,kw OR “Radiation Therapy”:ti,ab,kw OR “Radiation Therapies”:ti,ab,kw OR “Therapies, Radiation”:ti,ab,kw OR “Therapy, Radiation”:ti,ab,kw OR “Radiation Treatment”:ti,ab,kw OR “Radiation Treatments”:ti,ab,kw OR “Treatment, Radiation”:ti,ab,kw OR “Radiotherapy, Targeted”:ti,ab,kw OR “Radiotherapies, Targeted”:ti,ab,kw OR “Targeted Radiotherapies”:ti,ab,kw OR “Targeted Radiotherapy”:ti,ab,kw OR “Targeted Radiation Therapy”:ti,ab,kw OR “Radiation Therapies, Targeted”:ti,ab,kw OR “Targeted Radiation Therapies”:ti,ab,kw OR “Therapies, Targeted Radiation”:ti,ab,kw OR “Therapy, Targeted Radiation”:ti,ab,kw OR “Radiation Therapy, Targeted”:ti,ab,kw OR “Chemoradiotherapy”:ti,ab,kw OR “Chemoradiotherapies”:ti,ab,kw OR “Radiochemotherapy”:ti,ab,kw OR “Radiochemotherapies”:ti,ab,kw) |

# Supplementary Table 2. The definition of PFS and TTP in phase Ⅲ randomized controlled trials.

| Study | Endpoint type | Starting point | Event |
| --- | --- | --- | --- |
| Jett, 1990 | TTP | Randomization | Progression |
| Murray, 1993 | TTP | The first day of treatment | Progression |
| Gregor, 1997 | TTP | NA | NA |
| Turrisi, 1999 | PFS | NA | Progression, death |
| Takada, 2002 | PFS | Randomization | Progression, death |
| Schild, 2004 | PFS | NA | NA |
| Blackstock, 2005 | PFS | NA | NA |
| Giaccone, 2005 | PFS | NA | NA |
| McClay, 2005 | PFS | Randomization | Progression, death |
| Sculier, 2008 | TTP | Registration | Progression |
| Le Péchoux, 2009 | PFS | NA | NA |
| Sun, 2013* | PFS | Randomization | Progression, death |
| Kubota, 2014 | PFS | Randomization | Progression, death |
| Faivre-Finn, 2017 | PFS | NA | NA |
| Bogart, 2021 | PFS | NA | NA |

*The PFS definition was required by email.

Abbreviations: NA, not available; PFS, progression free survival; TTP, time to progression.

# Supplementary Table 3. The definition of PFS and TTP in phase Ⅱ and retrospective studies.

| Study | Endpoint type | Starting point | Event |
| --- | --- | --- | --- |
| *Phase Ⅱ randomized controlled trial (n=4)* | | | |
| Grønberg, 2016 | PFS | Randomization | Progression, death |
| Grønberg, 2021 | PFS | NA | NA |
| Peters, 2021 | PFS | Randomization | Progression, death |
| Qiu, 2021 | PFS | Randomization | Progression, death |
| *Single-arm phase Ⅱ study (n=8)* | | | |
| Hügli, 2000 | PFS | The first day of treatment | Progression, death |
| Thomas, 2001 | PFS | NA | NA |
| Ettinger, 2005 | PFS | Registration | Progression, death |
| Yilmaz, 2010 | PFS | The first day of treatment | Progression, death |
| CALGB 39808, 2013 | PFS | The first day of treatment | Progression, death |
| CALGB 30002, 2013 | PFS | The first day of treatment | Progression, death |
| CALGB 30206, 2013 | PFS | The first day of treatment | Progression, death |
| Xia, 2015 | PFS | NA | NA |
| *Retrospective study (n=10)* | | | |
| Kamath, 1998 | PFS | The first day of treatment | NA |
| Khanfir, 2011 | PFS | Diagnosis | Progression, death |
| Han, 2012 | PFS | The first day of treatment | Progression, death |
| Wang, 2015 | TTP | Diagnosis | Progression |
| Morimoto, 2016 | PFS | NA | Progression, death |
| Zhang, 2017 | PFS | The first day of treatment | Progression, death |
| Jeong, 2020 | PFS | The first day of treatment | Progression, death |
| Zayed, 2020 | PFS | Diagnosis | Progression, death |
| Atci, 2021 | TTP | Diagnosis | Progression |
| Doshita, 2021 | PFS | The first day of treatment | Progression, death |

Abbreviations: NA, not available; PFS, progression free survival; TTP, time to progression.

# Supplementary Table 4. The assessment of risk of bias in phase Ⅲ, Ⅱ randomized controlled trials by the Cochrane Collaboration tool.

| Trial | Bias | Risk of bias | Support for judgement |
| --- | --- | --- | --- |
| *Phase Ⅲ* |  |  |  |
| Jett, 1990 | Random sequence generation (selection bias) | Unclear risk | Insufficient information about the sequence generation process available to permit a judgement of ‘low risk’ or ‘high risk’. |
|  | Allocation concealment (selection bias) | Unclear risk | The method of concealment is not described to allow a definite judgement |
|  | Blinding of participants and personnel (performance bias) | Low risk | Open label. But outcome is not likely to be influenced by lack of blinding. |
|  | Blinding of outcome assessment (detection bias) | Low risk | Open label. But outcome is not likely to be influenced by lack of blinding. |
|  | Incomplete outcome data (attrition bias) | Unclear risk | Twelve patients (5%) were ineligible for study: five patients had insufficient material for pathologic evaluation, three patients had non-small-cell histology, three patients had extensive-stage disease, and one patient had the indicator lesion totally resected. |
|  | Selective reporting (reporting bias) | Low risk | All of prespecified outcomes were reported. |
|  | Other bias | Low risk | The study appeared to be free of other sources of bias. |
| Murray, 1993 | Random sequence generation (selection bias) | Unclear risk | Insufficient information about the sequence generation process available to permit a judgement of ‘low risk’ or ‘high risk’. |
|  | Allocation concealment (selection bias) | Unclear risk | The method of concealment is not described to allow a definite judgement |
|  | Blinding of participants and personnel (performance bias) | Low risk | Open label. But outcome is not likely to be influenced by lack of blinding. |
|  | Blinding of outcome assessment (detection bias) | Low risk | Open label. But outcome is not likely to be influenced by lack of blinding. |
|  | Incomplete outcome data (attrition bias) | Low risk | Insufficient reporting of attrition to permit a judgement of ‘low risk’ or ‘high risk’. |
|  | Selective reporting (reporting bias) | Low risk | All of prespecified outcomes were reported. |
|  | Other bias | Low risk | The study appeared to be free of other sources of bias. |
| Gregor, 1997 | Random sequence generation (selection bias) | Unclear risk | Treatment was assigned randomly by the EORTC Data Centre using the minimization technique |
|  | Allocation concealment (selection bias) | Unclear risk | Central randomization, participants and investigators can’t foresee assignment. |
|  | Blinding of participants and personnel (performance bias) | Low risk | Open label. But outcome is not likely to be influenced by lack of blinding. |
|  | Blinding of outcome assessment (detection bias) | Low risk | Open label. But outcome measurement is not likely to be influenced by lack of blinding. |
|  | Incomplete outcome data (attrition bias) | Unclear risk | Insufficient reporting of attrition to permit a judgement of ‘low risk’ or ‘high risk’ |
|  | Selective reporting (reporting bias) | Low risk | All of prespecified outcomes were reported. |
|  | Other bias | Low risk | The study appeared to be free of other sources of bias. |
| Turrisi, 1999 | Random sequence generation (selection bias) | Low risk | Patients were randomized according to a permuted-block scheme. |
|  | Allocation concealment (selection bias) | Unclear risk | The method of concealment is not described to allow a definite judgement |
|  | Blinding of participants and personnel (performance bias) | Low risk | Open label. But outcome is not likely to be influenced by lack of blinding. |
|  | Blinding of outcome assessment (detection bias) | Low risk | Open label. But outcome measurement is not likely to be influenced by lack of blinding. |
|  | Incomplete outcome data (attrition bias) | Low risk | All randomized patients were included in the analysis |
|  | Selective reporting (reporting bias) | Low risk | All of prespecified outcomes were reported. |
|  | Other bias | Low risk | The study appeared to be free of other sources of bias. |
| Takada, 2002 | Random sequence generation (selection bias) | Low risk | Randomization was performed centrally using the minimization method. |
|  | Allocation concealment (selection bias) | Low risk | Central randomization, participants and investigators can’t foresee assignment. |
|  | Blinding of participants and personnel (performance bias) | Low risk | Open label. But outcome is not likely to be influenced by lack of blinding. |
|  | Blinding of outcome assessment (detection bias) | Low risk | Open label. But outcome measurement is not likely to be influenced by lack of blinding. |
|  | Incomplete outcome data (attrition bias) | Low risk | All randomized patients were included in the analysis |
|  | Selective reporting (reporting bias) | Low risk | All of prespecified outcomes were reported. |
|  | Other bias | Low risk | The study appeared to be free of other sources of bias. |
| Schild, 2004 | Random sequence generation (selection bias) | Unclear risk | Insufficient information about the sequence generation process available to permit a judgement of ‘low risk’ or ‘high risk’. |
|  | Allocation concealment (selection bias) | Unclear risk | The method of concealment is not described to allow a definite judgement |
|  | Blinding of participants and personnel (performance bias) | Low risk | Open label. But outcome is not likely to be influenced by lack of blinding. |
|  | Blinding of outcome assessment (detection bias) | Low risk | Open label. But outcome measurement is not likely to be influenced by lack of blinding. |
|  | Incomplete outcome data (attrition bias) | Low risk | All randomized patients were included in the analysis |
|  | Selective reporting (reporting bias) | Low risk | All of prespecified outcomes were reported. |
|  | Other bias | Low risk | The study appeared to be free of other sources of bias. |
| Blackstock, 2005 | Random sequence generation (selection bias) | Unclear risk | Insufficient information about the sequence generation process available to permit a judgement of ‘low risk’ or ‘high risk’. |
|  | Allocation concealment (selection bias) | Unclear risk | The method of concealment is not described to allow a definite judgement |
|  | Blinding of participants and personnel (performance bias) | Low risk | Open label. But outcome is not likely to be influenced by lack of blinding. |
|  | Blinding of outcome assessment (detection bias) | Low risk | Open label. But outcome measurement is not likely to be influenced by lack of blinding. |
|  | Incomplete outcome data (attrition bias) | Low risk | 4/114 patients were excluded after randomization because of extensive disease: 1 patient in the continuous RT group and 3 patients in spilt RT group. Comment: similar reasons, unlikely to influence outcome. |
|  | Selective reporting (reporting bias) | Low risk | All of prespecified outcomes were reported. |
|  | Other bias | Low risk | The study appeared to be free of other sources of bias. |
| Giaccone, 2005 | Random sequence generation (selection bias) | Low risk | The randomization was performed by using the minimization technique |
|  | Allocation concealment (selection bias) | Low risk | Central randomization, participants and investigators can’t foresee assignment. |
|  | Blinding of participants and personnel (performance bias) | Low risk | Open label. But outcome is not likely to be influenced by lack of blinding. |
|  | Blinding of outcome assessment (detection bias) | Low risk | Open label. But outcome measurement is not likely to be influenced by lack of blinding. |
|  | Incomplete outcome data (attrition bias) | Low risk | All randomized patients were included in the analysis |
|  | Selective reporting (reporting bias) | Low risk | All of prespecified outcomes were reported. |
|  | Other bias | Low risk | The study appeared to be free of other sources of bias. |
| McClay, 2005 | Random sequence generation (selection bias) | Unclear risk | Insufficient information about the sequence generation process available to permit a judgement of ‘low risk’ or ‘high risk’. |
|  | Allocation concealment (selection bias) | Low risk | Central randomization, participants and investigators can’t foresee assignment. |
|  | Blinding of participants and personnel (performance bias) | Low risk | Open label. But outcome is not likely to be influenced by lack of blinding. |
|  | Blinding of outcome assessment (detection bias) | Low risk | Open label. But outcome measurement is not likely to be influenced by lack of blinding. |
|  | Incomplete outcome data (attrition bias) | Low risk | All randomized patients were included in the analysis |
|  | Selective reporting (reporting bias) | Low risk | All of prespecified outcomes were reported. |
|  | Other bias | Low risk | The study appeared to be free of other sources of bias. |
| Sculier, 2008 | Random sequence generation (selection bias) | Low risk | Randomisation was carried out using the minimisation technique |
|  | Allocation concealment (selection bias) | Low risk | Central randomization, participants and investigators can’t foresee assignment. |
|  | Blinding of participants and personnel (performance bias) | Low risk | Open label. But outcome is not likely to be influenced by lack of blinding. |
|  | Blinding of outcome assessment (detection bias) | Low risk | Open label. But outcome measurement is not likely to be influenced by lack of blinding. |
|  | Incomplete outcome data (attrition bias) | Unclear risk | Insufficient reporting of attrition to permit a judgement of ‘low risk’ or ‘high risk’ |
|  | Selective reporting (reporting bias) | Low risk | All of prespecified outcomes were reported. |
|  | Other bias | Low risk | The study appeared to be free of other sources of bias. |
| Le Péchoux, 2009 | Random sequence generation (selection bias) | Low risk | Eligible patients were randomized blindly by the data centre of the Institut Gustave Roussy using minimization. |
|  | Allocation concealment (selection bias) | Low risk | Central randomization, participants and investigators can’t foresee assignment. |
|  | Blinding of participants and personnel (performance bias) | Low risk | Open label. But outcome is not likely to be influenced by lack of blinding. |
|  | Blinding of outcome assessment (detection bias) | Low risk | Open label. But outcome measurement is not likely to be influenced by lack of blinding. |
|  | Incomplete outcome data (attrition bias) | Low risk | All randomized patients were included in the analysis |
|  | Selective reporting (reporting bias) | Low risk | All of prespecified outcomes were reported. |
|  | Other bias | Low risk | The study appeared to be free of other sources of bias. |
| Sun, 2013 | Random sequence generation (selection bias) | Low risk | Treatment was assigned using block randomization with variable block sizes. |
|  | Allocation concealment (selection bias) | Low risk | Central randomization, participants and investigators can’t foresee assignment. |
|  | Blinding of participants and personnel (performance bias) | Low risk | Open label. But outcome is not likely to be influenced by lack of blinding. |
|  | Blinding of outcome assessment (detection bias) | Low risk | Open label. But outcome measurement is not likely to be influenced by lack of blinding. |
|  | Incomplete outcome data (attrition bias) | Low risk | 3/222 patients were excluded after randomization: 2 patients in the early TRT because 1 had progression with malignant pleural effusion and the other withdrew consent before treatment commenced. 1 patient in the late TRT arm because the histologic diagnosis was changed to lymphoma after randomization.Comment: similar numbers, unlikely to influence outcome. |
|  | Selective reporting (reporting bias) | Low risk | All of prespecified outcomes were reported. |
|  | Other bias | Low risk | The study appeared to be free of other sources of bias. |
| Kubota, 2014 | Random sequence generation (selection bias) | Low risk | Eligible patients were randomly assigned in a 1:1 ratio to two groups |
|  | Allocation concealment (selection bias) | Low risk | Central randomization, participants and investigators can’t foresee assignment |
|  | Blinding of participants and personnel (performance bias) | Low risk | Open label. But outcome is not likely to be influenced by lack of blinding. |
|  | Blinding of outcome assessment (detection bias) | Low risk | Open label. But outcome measurement is not likely to be influenced by lack of blinding. |
|  | Incomplete outcome data (attrition bias) | Low risk | Only 1 patient was excluded after randomization because of contralateral hilar node metastasis, which is unlikely to influence outcome. |
|  | Selective reporting (reporting bias) | Low risk | All of prespecified outcomes were reported. |
|  | Other bias | Low risk | The study appeared to be free of other sources of bias. |
| Faivre-Finn, 2017 | Random sequence generation (selection bias) | Low risk | Patients were randomly assigned (1:1) to one of the two treatment groups (twice-daily vs once-daily radiotherapy). The allocation method used was minimisation with a random element using a bespoke computer application. |
|  | Allocation concealment (selection bias) | Low risk | Allocation to treatment group was done by phone call or fax from the recruiting centre. |
|  | Blinding of participants and personnel (performance bias) | Low risk | Open label. But outcome is not likely to be influenced by lack of blinding. |
|  | Blinding of outcome assessment (detection bias) | Low risk | Open label. But outcome measurement is not likely to be influenced by lack of blinding. |
|  | Incomplete outcome data (attrition bias) | Low risk | 4/574 patients were excluded after randomization because lost to follow-up: 3 in once-daily group and 1 in twice daily group. Comment: similar reasons, unlikely to influence outcome. |
|  | Selective reporting (reporting bias) | Low risk | All of prespecified outcomes were reported. |
|  | Other bias | Low risk | The study appeared to be free of other sources of bias. |
| Bogart, 2021 | Random sequence generation (selection bias) | Unclear risk | Insufficient information about the sequence generation process available to permit a judgement of ‘low risk’ or ‘high risk’. |
|  | Allocation concealment (selection bias) | Unclear risk | The method of concealment is not described to allow a definite judgement |
|  | Blinding of participants and personnel (performance bias) | Low risk | Open label. But outcome is not likely to be influenced by lack of blinding. |
|  | Blinding of outcome assessment (detection bias) | Low risk | Open label. But outcome measurement is not likely to be influenced by lack of blinding. |
|  | Incomplete outcome data (attrition bias) | Unclear risk | Insufficient reporting of attrition to permit a judgement of ‘low risk’ or ‘high risk’ |
|  | Selective reporting (reporting bias) | Low risk | All of prespecified outcomes were reported. |
|  | Other bias | Low risk | The study appeared to be free of other sources of bias. |
| *Excluded* |  |  |  |
| Maurer, 1997 | Random sequence generation (selection bias) | Unclear risk | Insufficient information about the sequence generation process available to permit a judgement of ‘low risk’ or ‘high risk’. |
|  | Allocation concealment (selection bias) | Unclear risk | The method of concealment is not described to allow a definite judgement |
|  | Blinding of participants and personnel (performance bias) | Low risk | Open label. But outcome is not likely to be influenced by lack of blinding. |
|  | Blinding of outcome assessment (detection bias) | Low risk | Open label. But outcome measurement is not likely to be influenced by lack of blinding. |
|  | Incomplete outcome data (attrition bias) | Low risk | 22/369 patients were ineligible because they did't receive protocol treatment. Comment: similar reasons, unlikely to influence outcome. |
|  | Selective reporting (reporting bias) | High risk | Chemotherapy course duration was modified from eight cycles to five cycles after 179 patients were enrolled. As a result, the patients were divided into two subgroup. The PFS analysis of combined total data set was not reported. |
|  | Other bias | Low risk | The study appeared to be free of other sources of bias. |
| *Phase Ⅱ* |  |  |  |
| Grønberg 2016 | Random sequence generation (selection bias) | Unclear risk | Insufficient information about the sequence generation process available to permit a judgement of ‘low risk’ or ‘high risk’. |
|  | Allocation concealment (selection bias) | Unclear risk | The method of concealment is not described to allow a definite judgement |
|  | Blinding of participants and personnel (performance bias) | Low risk | Open label. But outcome is not likely to be influenced by lack of blinding. |
|  | Blinding of outcome assessment (detection bias) | Low risk | Open label. But outcome measurement is not likely to be influenced by lack of blinding. |
|  | Incomplete outcome data (attrition bias) | Unclear risk | Insufficient reporting of attrition to permit a judgement of ‘low risk’ or ‘high risk’ |
|  | Selective reporting (reporting bias) | Low risk | All of prespecified outcomes were reported. |
|  | Other bias | Low risk | The study appeared to be free of other sources of bias. |
| Grønberg 2021 | Random sequence generation (selection bias) | Low risk | using a randomization module in an electronic clinical trial management system |
|  | Allocation concealment (selection bias) | Low risk | Both block sizes and allocation sequences in each block were generated by the system and masked to investigators. |
|  | Blinding of participants and personnel (performance bias) | Low risk | Open label. But outcome is not likely to be influenced by lack of blinding. |
|  | Blinding of outcome assessment (detection bias) | Low risk | Open label. But outcome measurement is not likely to be influenced by lack of blinding. |
|  | Incomplete outcome data (attrition bias) | Low risk | All randomized patients were included in the analysis |
|  | Selective reporting (reporting bias) | Low risk | All of prespecified outcomes were reported. |
|  | Other bias | Low risk | The study appeared to be free of other sources of bias. |
| Peters, 2021 | Random sequence generation (selection bias) | Low risk | Patients were randomized according to blocked stratified randomization. |
|  | Allocation concealment (selection bias) | Unclear risk | The method of concealment is not described to allow a definite judgement |
|  | Blinding of participants and personnel (performance bias) | Low risk | Open label. But outcome is not likely to be influenced by lack of blinding. |
|  | Blinding of outcome assessment (detection bias) | Low risk | Open label. But outcome measurement is not likely to be influenced by lack of blinding. |
|  | Incomplete outcome data (attrition bias) | Low risk | All randomized patients were included in the analysis |
|  | Selective reporting (reporting bias) | Low risk | All of prespecified outcomes were reported. |
|  | Other bias | Low risk | The study appeared to be free of other sources of bias. |
| Qiu 2021 | Random sequence generation (selection bias) | Low risk | Random assignment was done by a computer-generated random number code. |
|  | Allocation concealment (selection bias) | Unclear risk | The method of concealment is not described to allow a definite judgement |
|  | Blinding of participants and personnel (performance bias) | Low risk | Open label. But outcome is not likely to be influenced by lack of blinding. |
|  | Blinding of outcome assessment (detection bias) | Low risk | Open label. But outcome measurement is not likely to be influenced by lack of blinding. |
|  | Incomplete outcome data (attrition bias) | Unclear risk | Insufficient reporting of attrition to permit a judgement of ‘low risk’ or ‘high risk’ |
|  | Selective reporting (reporting bias) | Low risk | All of prespecified outcomes were reported. |
|  | Other bias | Low risk | The study appeared to be free of other sources of bias. |
| Hu 2012  (excluded) | Random sequence generation (selection bias) | Unclear risk | Insufficient information about the sequence generation process available to permit a judgement of ‘low risk’ or ‘high risk’. |
|  | Allocation concealment (selection bias) | Unclear risk | The method of concealment is not described to allow a definite judgement |
|  | Blinding of participants and personnel (performance bias) | Low risk | Open label. But outcome is not likely to be influenced by lack of blinding. |
|  | Blinding of outcome assessment (detection bias) | Low risk | Open label. But outcome measurement is not likely to be influenced by lack of blinding. |
|  | Incomplete outcome data (attrition bias) | Low risk | Only 1 patient was excluded after randomization because of a second primary small cell lung cancer, which is unlikely to influence outcome. |
|  | Selective reporting (reporting bias) | Low risk | All of prespecified outcomes were reported. |
|  | Other bias | High risk | Interim analysis. The calculated simple sizes are 504, but the interim analysis only included 85 patients. We believe the insufficient sample size would introduce bias and lead to unreliable results. |

# Supplementary Table 5. The assessment of risk of bias in single-arm phase Ⅱ, and retrospective studies by the Newcastle – Ottawa quality assessment scale for cohort study.

| Study | Selection | | | | Comparability | | Outcome | | | Total score |
| --- | --- | --- | --- | --- | --- | --- | --- | --- | --- | --- |
|  | Representativeness of the exposed cohort | Selection of the non-exposed cohort | Ascertainment of exposure | Demonstration that outcome of interest was not present at start of study | Comparability of cohorts on the basis of the design or analysis | | Assessment of outcome | Median follow-up greater than 1 year | Adequacy of follow-up of cohorts |  |
|  |  |  |  |  | Age | Smoking history |  |  |  |  |
| *Single-arm phase II study (n=8)* | | | | | | | | | | |
| Hügli, 2000 | 1 | 0 | 1 | 1 | 0 | 0 | 1 | 1 | 1 | 6 |
| Thomas, 2001 | 1 | 0 | 1 | 1 | 0 | 0 | 1 | 1 | 1 | 6 |
| Ettinger, 2005 | 1 | 0 | 1 | 1 | 0 | 0 | 1 | 1 | 1 | 6 |
| Yilmaz, 2010 | 1 | 0 | 1 | 1 | 0 | 0 | 1 | 1 | 1 | 6 |
| CALGB 39808, 2013 | 1 | 0 | 1 | 1 | 0 | 0 | 1 | 1 | 1 | 6 |
| CALGB 30002, 2013 | 1 | 0 | 1 | 1 | 0 | 0 | 1 | 1 | 1 | 6 |
| CALGB 30206, 2013 | 1 | 0 | 1 | 1 | 0 | 0 | 1 | 1 | 1 | 6 |
| Xia, 2015 | 1 | 0 | 1 | 1 | 0 | 0 | 1 | 1 | 1 | 6 |
| *Retrospective study (n=11)* | | | | | | | | | | |
| Kamath, 1998 | 1 | 0 | 1 | 1 | 0 | 0 | 1 | 1 | 1 | 6 |
| Khanfir, 2011 | 1 | 0 | 1 | 1 | 0 | 0 | 1 | 1 | 1 | 6 |
| Han, 2012 | 1 | 0 | 1 | 1 | 1 | 0 | 1 | 1 | 1 | 7 |
| Han, 2015(excluded) | 0 | 0 | 1 | 1 | 1 | 0 | 1 | 1 | 0 | 5 |
| Wang, 2015 | 1 | 1 | 1 | 1 | 0 | 1 | 1 | 1 | 1 | 8 |
| Morimoto, 2016 | 1 | 0 | 1 | 1 | 0 | 0 | 1 | 1 | 1 | 6 |
| Zhang, 2017 | 1 | 1 | 1 | 1 | 1 | 1 | 1 | 1 | 1 | 9 |
| Jeong, 2020 | 1 | 0 | 1 | 1 | 0 | 0 | 1 | 1 | 1 | 6 |
| Zayed, 2020 | 1 | 1 | 1 | 1 | 1 | 1 | 1 | 1 | 1 | 9 |
| Atci, 2021 | 1 | 0 | 1 | 1 | 0 | 0 | 1 | 1 | 1 | 6 |
| Doshita, 2021 | 1 | 0 | 1 | 1 | 0 | 0 | 1 | 1 | 1 | 6 |

# Supplementary Table 6. Four subgroups for phase Ⅲ randomized controlled trials.

| Subgroup | Study |
| --- | --- |
| Different chemotherapy | Jett, 1990 |
|  | Sculier, 2008 |
|  | Kubota, 2014 |
| Novel drug | Giaccone, 2005 |
|  | McClay, 2005 |
| Early vs late radiotherapy | Murray, 1993 |
|  | Takada, 2002 |
|  | Sun, 2013 |
| Different radiotherapy model | Gregor, 1997 |
|  | Turrisi, 1999 |
|  | Schild, 2004 |
|  | Blackstock, 2005 |
|  | Le Péchoux, 2009 |
|  | Faivre-Finn, 2017 |
|  | Bogart, 2021 |

# Supplementary Figure 1. Flow diagram of research selection.


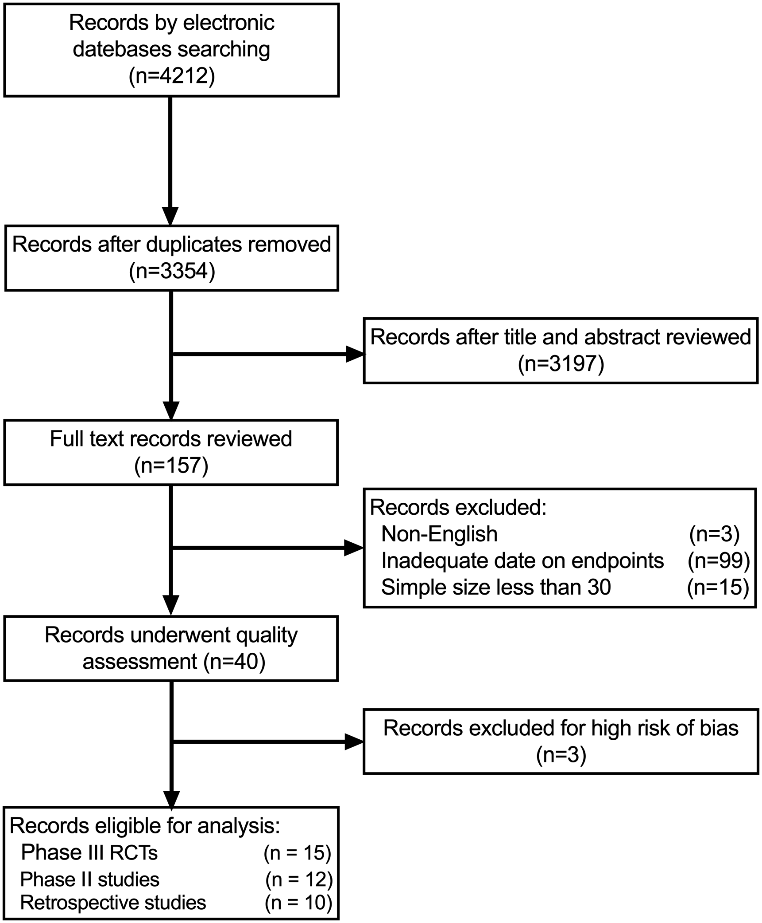


Abbreviation: RCTs, randomized controlled trials

# Supplementary Figure 2. Sensitivity analysis across different settings


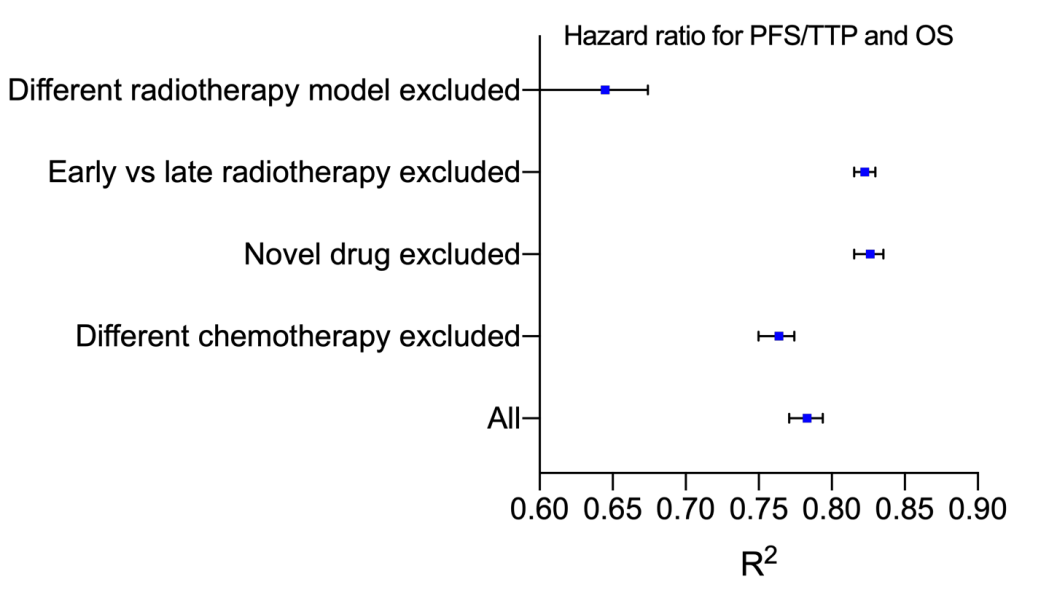

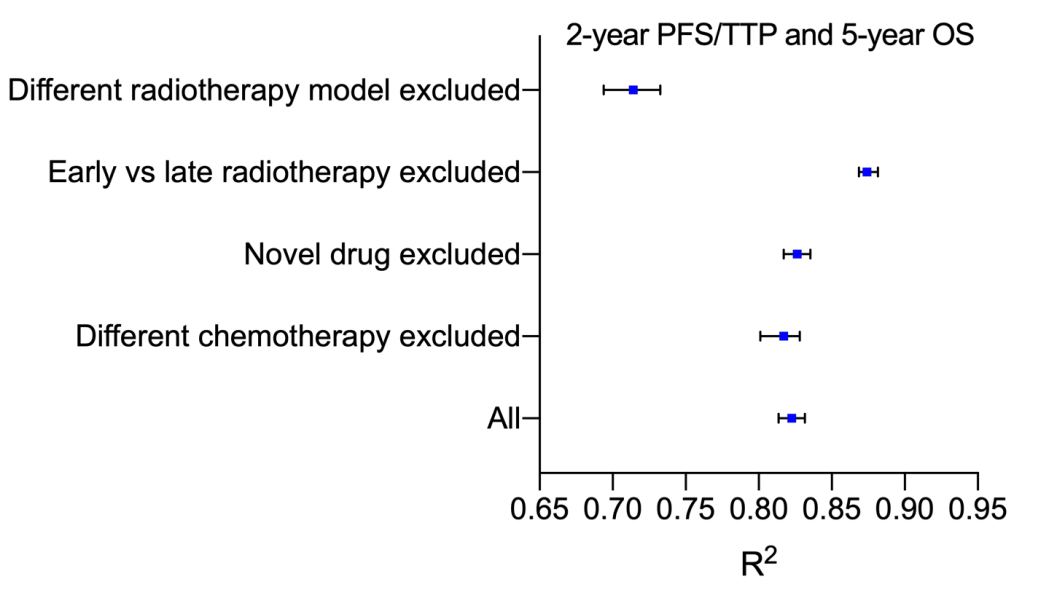


B

A


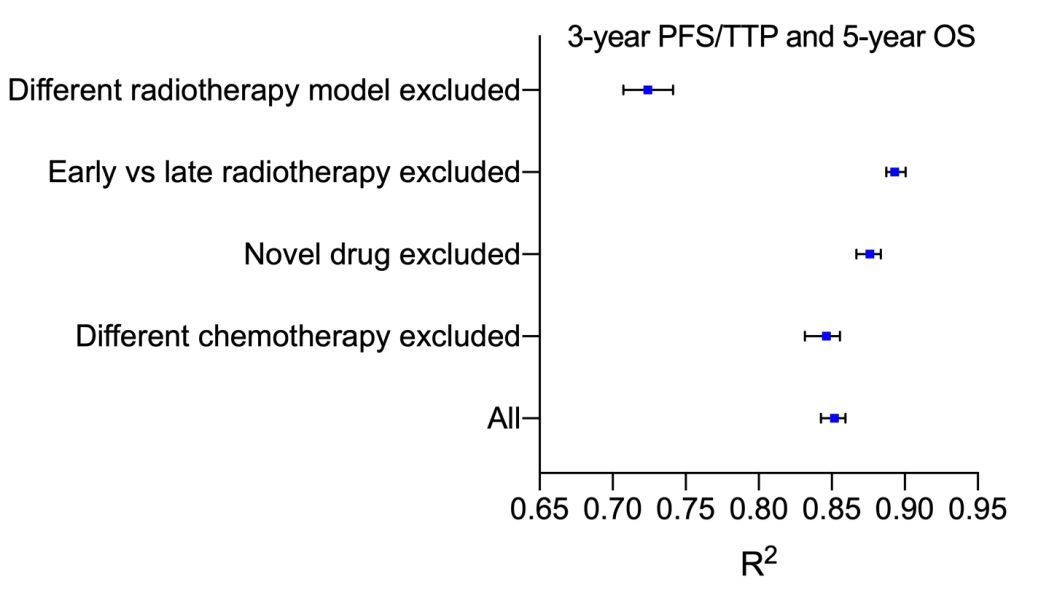

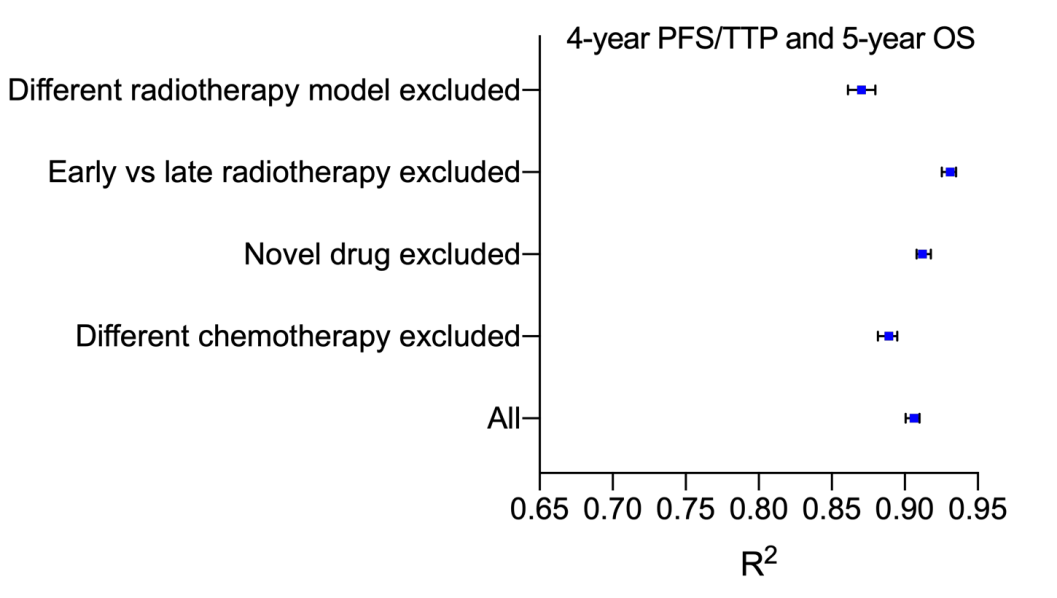


D

C


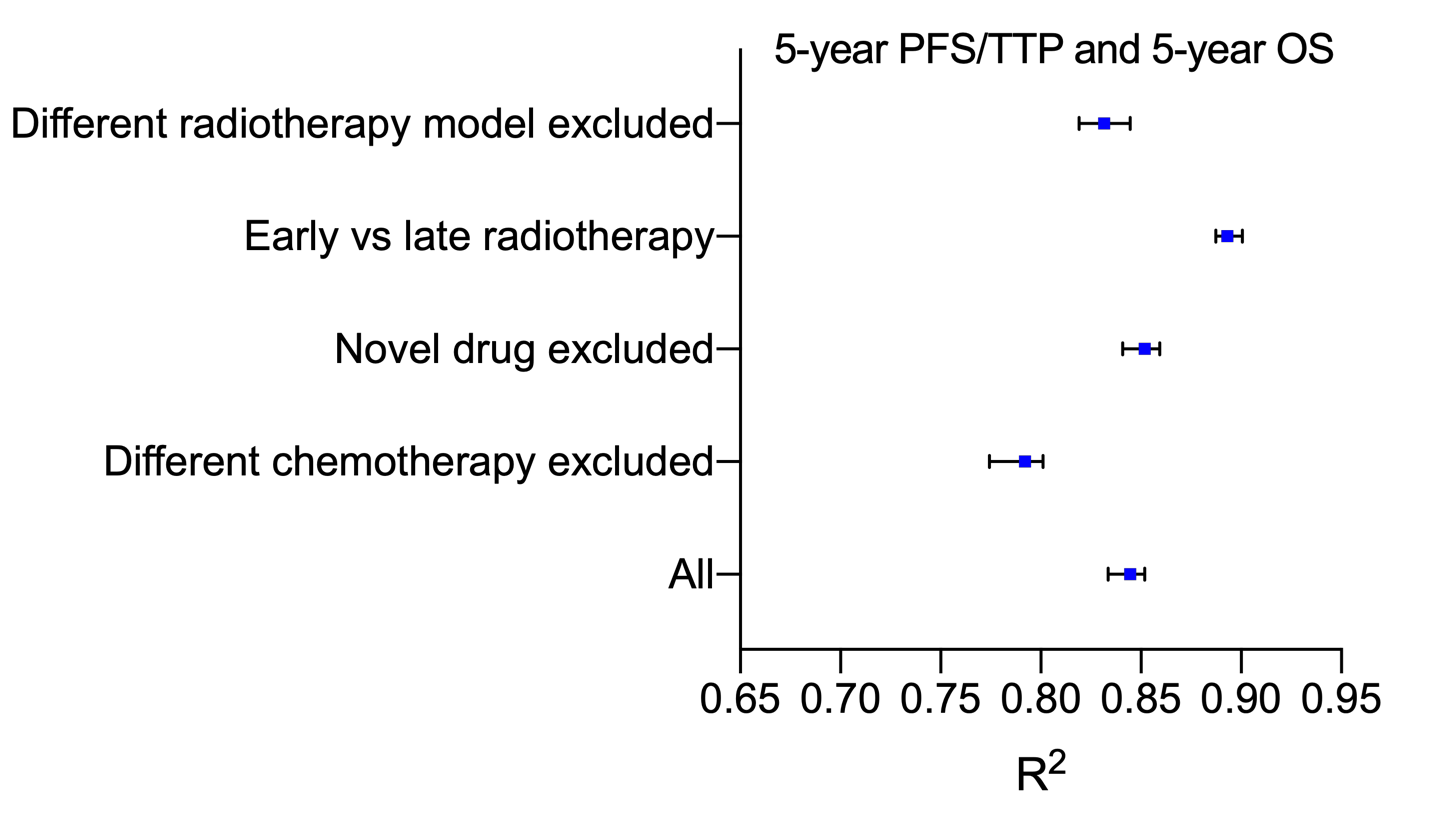


E

# Supplementary Figure 3. Plot of R^2^ and PFS/TTP year.

Abbreviations: PFS, progression free survival; TTP, time to progression
